# Vitamin D Deficiency Is Inversely Associated with Homeostatic Model Assessment of Insulin Resistance

**DOI:** 10.3390/nu13124358

**Published:** 2021-12-03

**Authors:** Shamaila Rafiq, Per Bendix Jeppesen

**Affiliations:** Department of Clinical Medicine, Aarhus University, 8200 Aarhus, Denmark

**Keywords:** Homeostatic Model Assessment of Insulin Resistance, HOMA-IR, vitamin D deficiency, type 2 diabetes (T2D), body mass index, BMI, insulin resistance

## Abstract

The study was conducted to comprehensively assess the association of the concentration of vitamin D in the blood and insulin resistance in non-diabetic subjects. The objective was to pool the results from all observational studies from the beginning of 1980 to August 2021. PubMed, Medline and Embase were systematically searched for the observational studies. Filters were used for more focused results. A total of 2248 articles were found after raw search which were narrowed down to 32 articles by the systematic selection of related articles. Homeostatic Model Assessment of Insulin Resistance (HOMAIR) was used as the measure of insulin resistance and correlation coefficient was used as a measure of the relationship between vitamin D levels and the insulin resistance. Risk of bias tables and summary plots were built using Revman software version 5.3 while Comprehensive meta-analysis version 3 was used for the construction of forest plot. The results showed an inverse association between the status of vitamin D and insulin resistance (r = −0.217; 95% CI = −0.161 to −0.272; *p* = 0.000). A supplement of vitamin D can help reduce the risk of insulin resistance; however further studies, like randomized controlled trials are needed to confirm the results.

## 1. Introduction

A secosteroid hormone, vitamin D has a variety of pathologic and physiologic functions in the human body. In addition to its function in the bone metabolism because of its involvement in the phosphate and calcium absorption [1], this vitamin is recently understood to have relationship with the prevention of the diseases, e.g., insulin resistance, type 2 diabetes (T2D), and cardiovascular disease [2,3,4].

The drive to write this review article is to explore the present understanding of the relationship of vitamin D status with insulin resistance. The insulin resistance is related with obesity, hormonal disorders and overnutrition [5,6]. Glucose homeostasis is normally regulated by insulin which directs the uptake of glucose in the cells [7]. An imbalance in the secretion of insulin or its action can cause metabolic disorders like hyperglycemia and disturbed regulation of lipoproteins, triglycerides and fatty acids. These irregulations can further complicate insulin homeostasis. Hypovitaminosis D is involved in the production of inflammatory cytokines, and an increase in inflammatory cytokines can be the cause of insulin resistance and eventually T2D. The anti-inflammatory effect of vitamin D may have a role in the improvement of insulin sensitivity in patients with relatively higher BMI [8,9].

One of the risk factors for insulin resistance is obesity. Insulin works to reduce the glucose concentration in the blood and is very important for the utilization of glucose [9,10]. In the liver, skeletal muscles and adipose tissues insulin binds to the receptors in the cell membrane and metabolic reactions occur to lower the glucose level. The target is achieved by multiple actions, e.g., by storing glucose in the liver, utilizing it in the adipose tissues and by regulating the genes related to glucose homeostasis, lipid synthesis, lipolysis and reducing the activity of pyruvate carboxylase which in turn reduces gluconeogenesis in the liver [11,12].

It has been observed that the risks for insulin resistance, T2D and hypovitaminosis D are almost the same irrespective of ethnicity but might be related to the sun exposure [13,14,15]. Other studies have confirmed that the seasonal variations in the status of insulin and vitamin D are correlated [16,17,18]. Obesity and vitamin D have been observed to be inversely associated in previous studies [19,20]. A daily dosage of 1200 IU of vitamin D for more than four months to obese children reduced the BMI significantly [21]. It has been observed that people with obesity have less exposure to sunlight, inadequate intake of vitamin D, rarely do exercise and have limited outdoor activities. On the other hand, being fat soluble, vitamin D can be sequestered in the adipose tissues which also explains its reduced bioavailability [22,23,24]. Vitamin D receptor (VDR) is expressed in the beta cells of the pancreas, where vitamin D binds to it, helping in the release of insulin secretion [25]. Vitamin D is directly involved in the expression of insulin receptor in muscles, adipose tissues and liver [26]. Research shows that vitamin D also protects against insulin resistance by up-regulating insulin receptors and increasing insulin sensitivity [27,28].

The meta-analysis conducted here was to see the association of vitamin D status and HOMA-IR. HOMA-IR represents the strength of insulin resistance. We used forest plot to see the correlation of vitamin D status and HOMA-IR. We assume that the vitamin D status is affected by the latitude, a meta-regression analysis was therefore performed to find out the effect of latitude on this correlation if any. We also performed the meta-regression analysis for the method of determination of vitamin D as well, for it could also have an effect on the relationship of vitamin D status and insulin resistance. This review examined the relationship of vitamin D with HOMA-IR, and sub-group analyses were conducted to see the effect of BMI on this relationship.

## 2. Materials and Methods

The articles were approved to be included in the current review if they were original observational studies, written in English and involved adult (at least 18 years of age) human beings. We excluded any commentaries, reports and editorials for this meta-analysis. The authors were contacted for lacked information if considered necessary. In addition to systematic search the related articles were hand searched for additional references. The search string for the study was developed taking into consideration the strategies for systematic meta-analyses search. The databases Embase, PubMed and Medline were searched for relevant articles using following search terms, “25 (OH) vitamin D”, “cholecalciferol”, “25 (OH) D”, “vitamin D” and “vitamin D3”in combination with “homeostasis model assessment of insulin resistance”, “HOMAIR”, “Insulin”, “fasting plasma insulin”, “Insulin resistance”, “HBA1C”, “type 2 diabetes”, “fasting plasma glucose”, “Insulin Sensitivity”, “Insulin Secretion”, “Metabolic syndrome”, “abdominal obesity”, “adiposity”, and “T2D”. The keywords search was conducted both as free keywords and combination (EMTREE in Embase, and MeSH in PubMed). The filters applied for the search were English language, human subjects and original articles. Endnote software was used to indicate duplicate entries. The records were then screened independently by authors for title and abstract. Finally, the full-length articles were assessed for eligibility. The discrepancies between the authors were resolved by reading the articles together again. The data were extracted from the studies finalized by the authors. The important data parts were collected to calculate the potential moderators and the effect sizes.

Subgroup and Moderator Analysis.

To assess the effect of BMI on the general relationship of vitamin D status and HOMA-IR the studies were divided in three groups of different BMI ranges (<20, 20–30 and >30) and subgroup analyses were performed where the data were enough to perform subgroup meta-analysis. Two studies did not mention the BMI therefore they were excluded from subgroup analysis. The values of moderators both qualitative (method of determination of vitamin D) and quantitative (latitude and BMI) were run to calculate R^2^.

### Statistical Analysis and Outcome Measures

The effect size was presented as correlation. If the correlation was not reported in the article the electronic spread sheet was used to convert the existing data to correlation. We selected random effect model for the calculation of meta-analysis and the outcome summary measures. The consistency and reliability were assessed by the estimates like I^2^ and τ^2^, respectively. The I^2^ describes the heterogeneity in percentage among studies. The publications were assessed for quality to account for: 1. Indirectness (compromised generalizability of results); 2. Inconsistency (unexplained heterogeneity between studies); 3. Publication bias (small number of participants) and 4. Imprecision (too long confidence intervals). Grades of Recommendation Assessment Development and Evaluation (GRADE) was used for quality assessment of the articles. Meta-analysis and meta-regression were performed using Comprehensive Meta-Analysis Version 3 (Biostat, Inc., Englewood, NJ, USA) while risk of bias (ROB) analysis was performed using Review Manager 5.3. (Cochrane Collaboration, Oxford, UK).

## 3. Results

**Figure 1:**
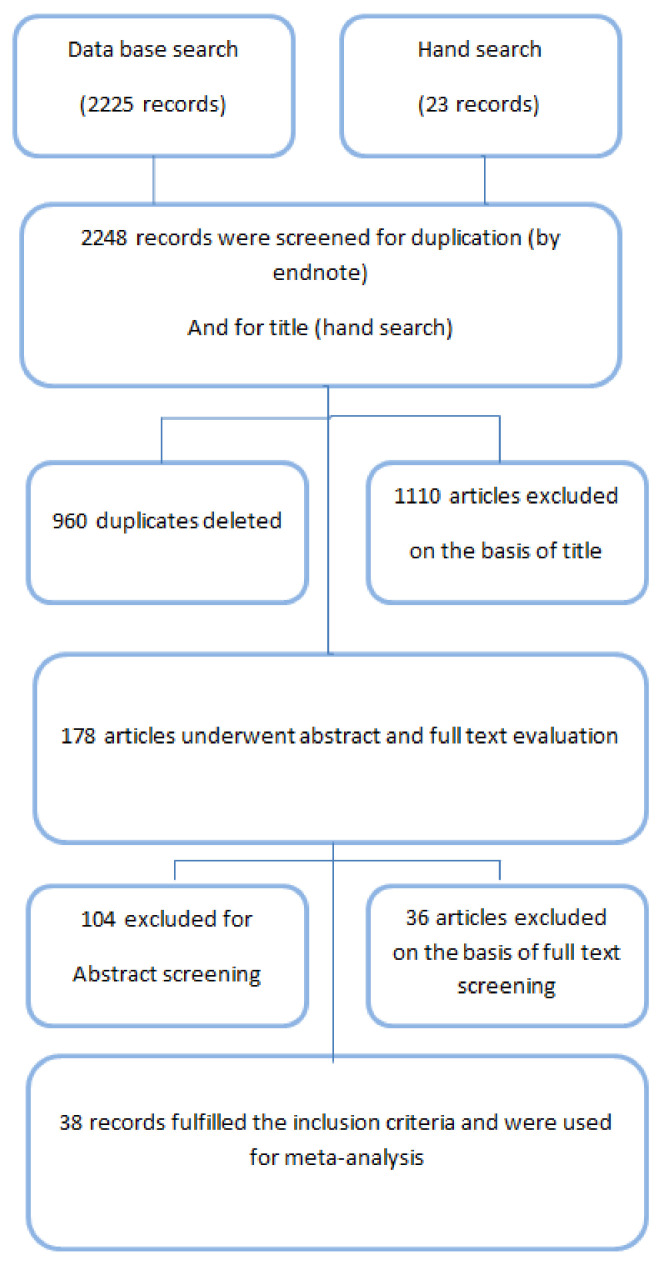
Flow sheet diagram of the selection of the articles.

Two thousand two hundred and twenty-five studies were collected electronically from Medline, Embase and PubMed and 23 entries were retrieved by hand search. Nine-hundred-and-sixty duplicate studies were identified by Endnote and were deleted. Eleven-hundred-and-ten articles were rejected on the basis of title. One hundred and seventy-eight references were selected for abstract assessment. On the basis of abstract 104 articles were rejected and the remaining 36 articles underwent full text evaluation. Thirty-eight studies were selected for meta-analysis (Figure 1).

### 3.1. Excluded Studies

Eight studies [29,30,31,32,33,34,35,36] were excluded because the outcome measures were unable to be converted to correlation coefficient. Seventeen studies [37,38,39,40,41,42,43,44,45,46,47,48,49,50,51,52,53] were excluded because the design of study was not compatible with the desired plan to be considered for inclusion. Eleven [54,55,56,57,58,59,60,61,62,63,64] studies were rejected because they did not deal the diabetic and non-diabetic subjects separately. Three articles [65,66,67] were excluded because the number of participants were not mentioned in each vitamin D quartile. Seven studies [68,69,70,71,72,73,74] were excluded because their full-length articles were not found.

### 3.2. Included Studies

**Figure 2:**
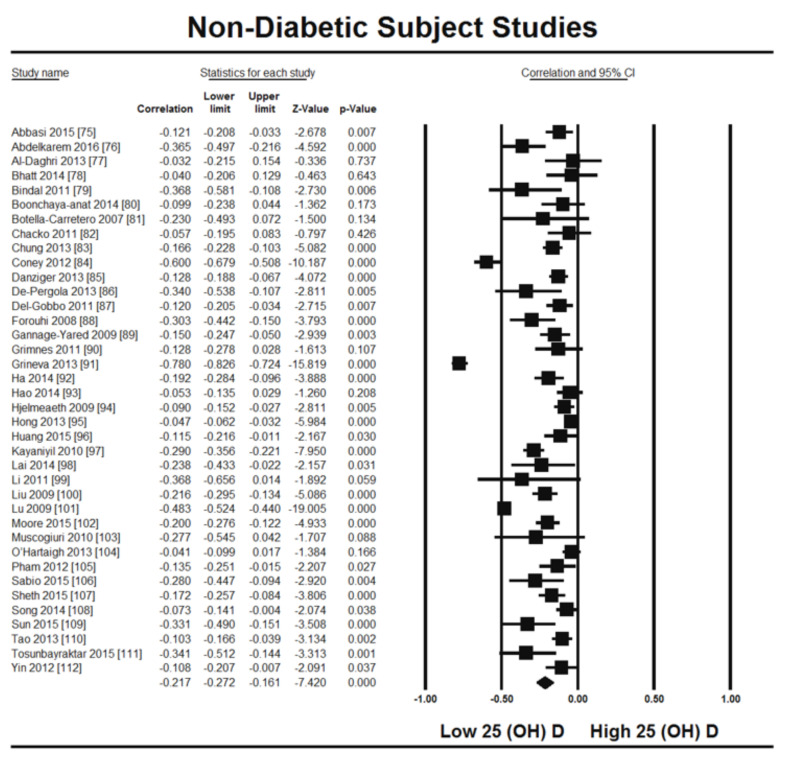
Forest plot showing the relationship of vitamin D status and HOMAIR [75,76,77,78,79,80,81,82,83,84,85,86,87,88,89,90,91,92,93,94,95,96,97,98,99,100,101,102,103,104,105,106,107,108,109,110,111,112], 95% confidence interval (CI) (I^2^= 94.4% *p* = 0.00) and correlation were calculated by using random effect model.

**Figure 3:**
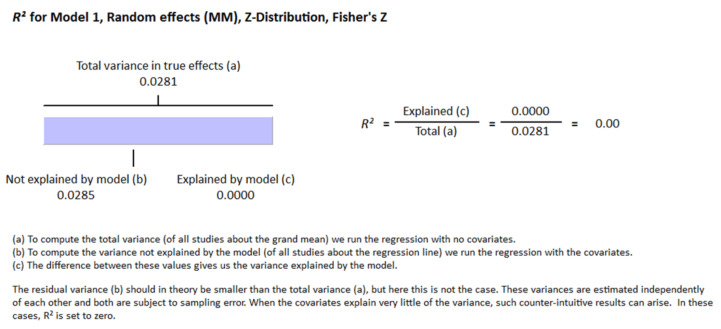
Meta regression analysis for the moderator latitude, R-Squared represents the contribution of latitude to the variability of correlation.

**Figure 4:**
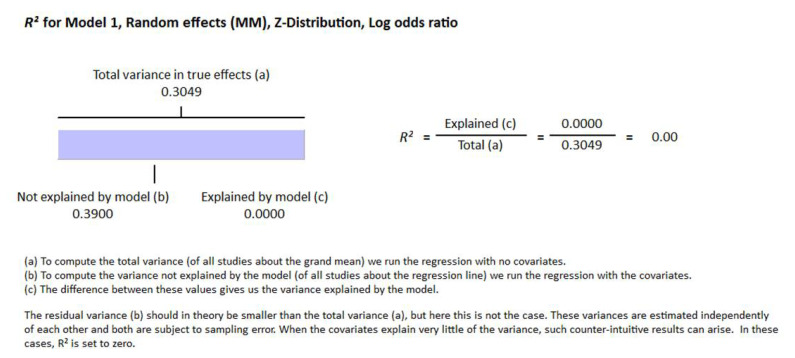
Meta regression analysis for the moderator method of determination of vitamin D, R-Squared represents the contribution of method of determination of vitamin D to the variability of correlation.

Thirty-two studies were included in this meta-analysis from 1980 to August 2021. All participants were adult and at least 18 years of age. The latitude ranges from 23 to 70 degrees for all studies. Different methods were used for the determination of vitamin D in different studies. Sixteen articles used radioimmunoassay (RIA), seven studies used chemiluminescence assay (CLIA), five studies used enzyme-linked immunosorbent assay (ELISA), four studies used liquid chromatography–mass spectrometry (LC-MS), three studies used electrochemiluminescence assay (ECLIA), one study used high-performance liquid chromatography (HPLC) for the vitamin D determination. Two research articles included in this meta-analysis did not mention the method of determination of vitamin D. We used random effect model for this meta-analysis because we used observational studies which potentially have more sources of variation. From this review it is evident that vitamin D status is inversely related with HOMA-IR in the non-diabetic group of the population (r = −0.217, 95% = −0.271 to −0.161, *p* = 0.000) (Figure 2). The correlation ranges from r = −0.03 to r = −0.78. We observed no heterogeneity in the correlation due to method of determination of vitamin D and latitude as evident from the meta-regression analysis (R^2^ = 0.000, *p* = 0.000). This means the relationship of vitamin status and HOMA-IR is independent of these two variables (Figure 3 and Figure 4).

**Figure 5:**
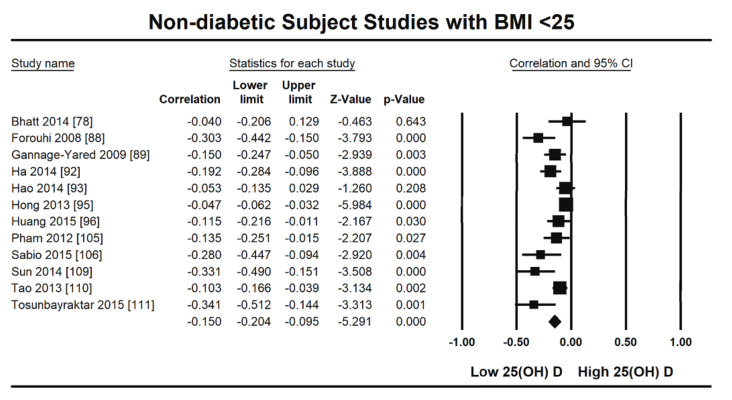
Non-diabetic subject studies for the lowest BMI quartile (18–25): Forest plot showing the relationship of vitamin D status and HOMAIR [78,88,89,92,93,95,96,105,106,109,110,111].

**Figure 6:**
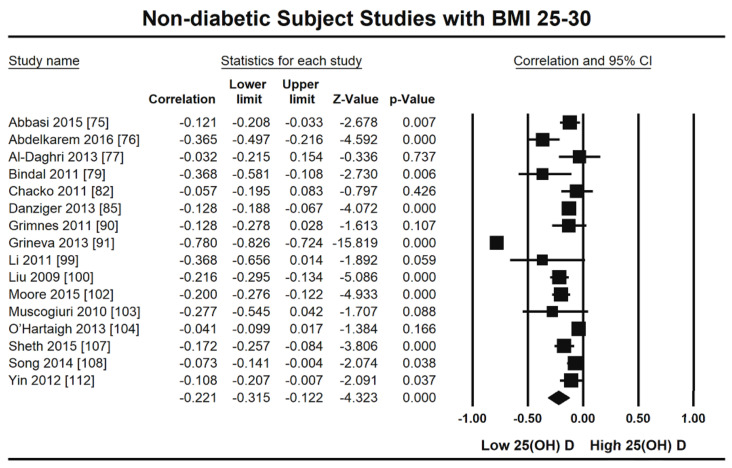
Non-diabetic subject studies for the medium BMI quartile (25–30): Forest plot showing the relationship of vitamin D status and HOMAIR [75,76,77,79,82,85,90,91,99,100,102,103,104,107,108,112].

**Figure 7:**
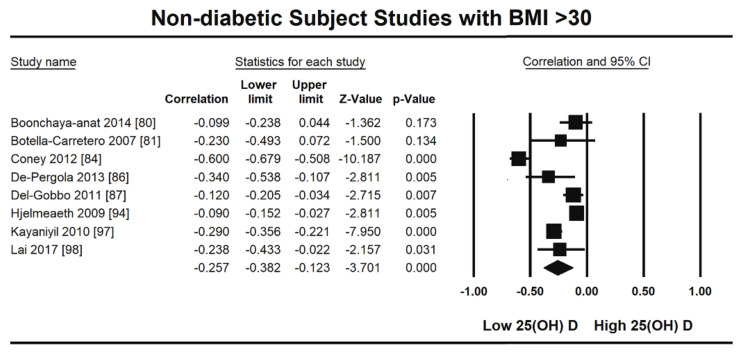
Non-diabetic subject studies for the highest BMI quartile (>30). Forest plot showing the relationship of vitamin D status and HOMAIR [80,81,84,86,87,94,97,98].

**Figure 8:**
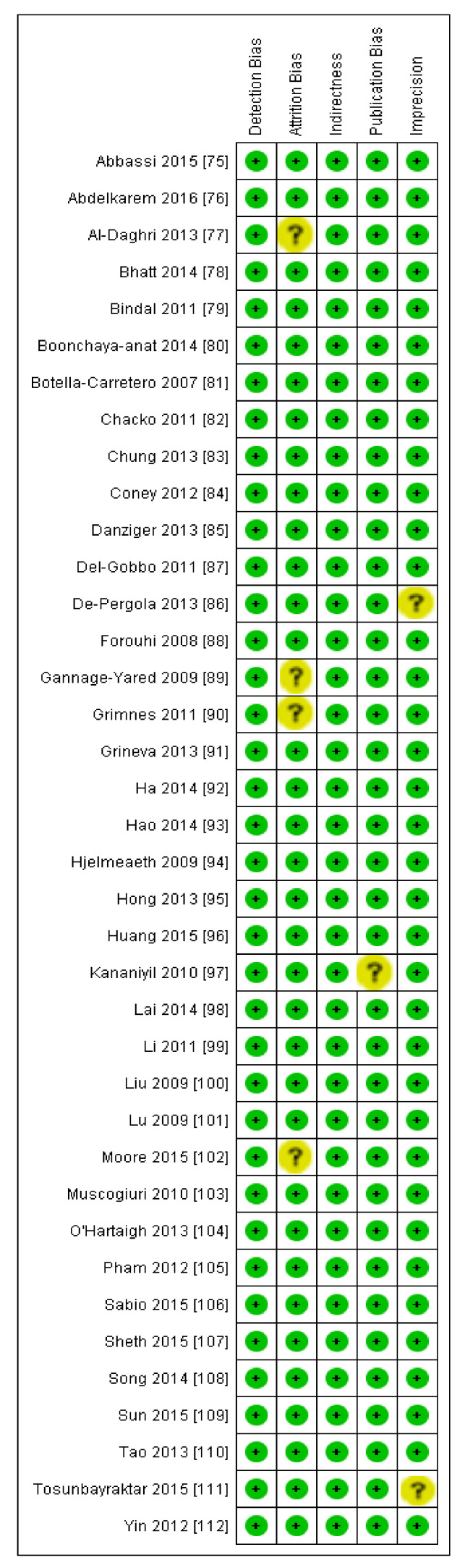
Risk of bias summary, data shown for individual studies (plus sign shows low ROB and question mark shows unknown ROB) [75,76,77,78,79,80,81,82,83,84,85,86,87,88,89,90,91,92,93,94,95,96,97,98,99,100,101,102,103,104,105,106,107,108,109,110,111,112].

**Figure 9:**
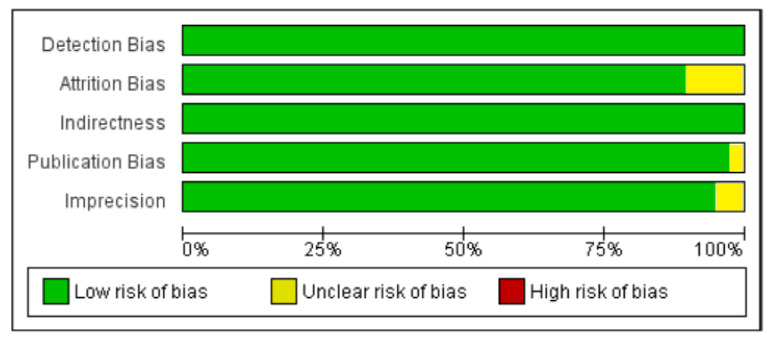
Author’s assessment of the risk of bias in non-diabetic subject studies: Data shown in percentages for all studies.

The summary and graph of GRADE (Grades of Recommendation, Assessment, Development and Evaluation) are shown in the figures (Figure 5 and Figure 6). The subgroup analysis based on different BMI quartiles showed a gradual increase in the strength of correlation (vitamin D and HOMA-IR) from lower to higher BMI quartiles. For instance, it was lowest for BMI less than 25 (r = −0.150, 95% = −0.204 to −0.095, *p* = 0.000) (Figure 5), moderate for BMI 25–30 (r = −0.221, 95% = −0.315 to −0.122, *p* = 0.000) (Figure 6) and highest for BMI more than 30 (r = −0.257, 95% = −0.382 to −0.123, *p* = 0.000) (Figure 7). The correlation was shown to be highly significant in the high BMI quartiles compared to the lower one. Generally, an inverse association has been observed between vitamin D status and HOMA-IR in all studies in this meta-analysis. However, four studies (Coney 2012, Grineva 2013, Li 2011 and Lu 2009) [84,91,99,101] showed higher correlation than the rest. Among these four studies Coney from USA and Grineva from Russia showed exceptionally high correlation, i.e., r = −0.6 and r = −0.78 while Li from UK and Lu from China showed moderately high correlation, i.e., r = −0.36 and r = −0.48 respectively. The GRADE assessment for this meta analysis is shown in Figure 8 and Figure 9.

## 4. Discussion

From this review it is observed that the level of vitamin D is inversely associated with HOMA-IR in non-diabetic subjects (r = −0.217, 95% = −0.271 to −0.161, *p* = 0.000) (Figure 2). The subgroup analysis based on different BMI quartiles showed a significantly measured increase in the power of correlation between vitamin D and HOMA-IR from lower (<25 BMI) to higher (>30 BMI) BMI quartile. For instance, it was lowest for BMI less than 25 (r = −0.150, 95% = −0.204 to −0.095, *p* = 0.000) (Figure 5), moderate for BMI 25–30 (r = −0.221, 95% = −0.315 to −0.122, *p* = 0.000) (Figure 6) and highest for BMI more than 30 (r = −0.257, 95% = −0.382 to −0.123, *p* = 0.000) (Figure 7). This correlation pattern might relate the coexistence of hypovitaminosis D and obesity in a large number of clinical disorders [113], most relevant here is insulin resistance [114]. It has been observed earlier that vitamin D has a direct impact on BMI [115] and a decrease of 1.3 nM/L of vitamin D can increase the BMI by 1 kg/m^2^ [116]. The gradual increase in the strength of relationship from lower to higher BMI quartile indicates that the synergistic effect of BMI and hypovitaminosis D might be the reason behind the development of insulin resistance.

It has been discovered earlier that the primary mediator of insulin resistance is abdominal adiposity which can deregulate the anti-diabetic hormone leptin [117]. High secretion of this hormone is related to insulin resistance. Some randomized controlled trials showed a decreased leptin level [118] and a reduced BMI [119] after high doses of vitamin D administration to insulin-resistant patients.

An extra need of insulin secretion compared to normal to maintain a normal level of glucose in the blood defines insulin resistance. The beta cells are exhausted by continuous insulin production and can lead to T2D. Insulin resistance can also lead to many other diseases like polycystic ovaries and non-alcoholic fatty liver disease (NAFL) [120,121,122]. The deficiency of vitamin D has been considered to be related to T2D previously [123,124,125]. Hypovitaminosis is also related with the development of nonalcoholic fatty liver disease. Vitamin D is a prohormone that has autocrine, paracrine and endocrine functions [126,127,128,129]. Hypovitaminosis D develops insulin resistance that progresses to type 2 diabetes and obesity [130]. Research shows that the progression of T2D and a severe hyperglycemic condition after carbohydrate consumption is reduced with the supplementation of vitamin D [131,132,133]. The evidence for the correlation of hypovitaminosis D and insulin resistance has been observed in a range of studies previously including our current and previous studies [134,135,136].

Vitamin D can be naturally synthesized from sun exposure to skin, UV-B rays emitted from the sun, and photosynthetically prepared vitamin D in the skin. The access to UV-B radiation has been scarce owing to many reasons, e.g., due to industrialization and use of concrete in the buildings, these rays are scattered and absorbed, and their strength is much reduced [137]. The irradiance of UV-B is also affected by industrial gases and O_3_ from the ozone layer, these gases are absorbed in the ultraviolet B region, and the UV-B irradiance is therefore compromised [138]. Moreover, ethnic trends in different populations like time of sun bath, skin color, and means of leisure, occupation, travel and food habits also determine the status of vitamin D produced naturally by the sun. The latitude therefore can have little or no effect as evident from the meta-regression analysis in our current studies and the studies conducted previously [136]. Living in low latitudes does not guarantee a good vitamin D status. Generally, there existed an inverse relationship between vitamin D status and HOMA-IR in all studies in this meta-analysis. However, four studies [84,91,99,101] showed higher correlation than the rest. Among these four studies Coney from USA and Grineva from Russia [84,91] showed exceptionally high correlation, i.e., r = −0.6 and r = −0.78 while Li from UK and Lu from China [99,101] showed moderately high correlation i.e., r = −0.36 and r = −0.48. Interestingly all participants from three of these studies [84,91,99] were females and the fourth study [101] included more females than males. The women from Russia showing highest correlation (r = −0.78) were in their late reproductive age, i.e., from 40–52 years. The American female population showing a little less correlation (r = −0.6) however includes most of the subjects with early reproductive age group 18–45 years. The study from UK showing a moderately high correlation (r = −0.36) included females aged 27–40 years, and the female participants in the Chinese study with a correlation of r = −0.48 were in the age group of 50–70. Apparently, it looks like the age group or the menopausal age range does not have an impact on the dependence of insulin resistance in the female population. Therefore, we can say that women show more dependency on vitamin D status for insulin resistance, and one of the major causes of insulin resistance in women might be hypovitaminosis D. Thus, vitamin D therapy in women might get better results for the correction of insulin resistance. However, this assumption needs to be investigated further.

The deficiency of vitamin D impairs glucose stimulated insulin secretion [139,140,141] from the beta cells and this impairment is restored by vitamin D supplementation [139,140,142,143]. The expression of vitamin D receptors (VDR) in the beta cells, the existence of vitamin D response elements (VDRE) in the promoter region of the insulin gene and the activation of the insulin gene by 1, 25 (OH) vitamin D give the indication that vitamin D might have a direct role in the secretion of insulin [144,145,146]. The beta cell function could therefore be corrected in the early stage of development of insulin resistance by vitamin D intervention. Vitamin D requires VDR for its functioning in different types of cells; however, the expression of VDR in different tissues depends on the presence of calcium and/or vitamin D or neither of them. It has been published earlier that vitamin D prompts the insulin secretion from beta cells and reduces insulin resistance in muscle, adipose tissues and liver [147,148,149]. Vitamin D acts at the transcription level as an epigenetic factor for many genes that increases insulin sensitivity. For instance, the expression of IRS (insulin receptor substrate) is increased by 2.4-fold by treatment with vitamin D in high-fat treated mice models. IRS protein is known for increasing insulin sensitivity in the target tissues [150]. We observed no heterogeneity in the correlation due to method of determination of vitamin D as evident from the meta-regression analysis (R^2^ = 0.000, *p* = 0.00).

Hypovitaminosis D and insulin resistance could be genetically inter-related. The glucose metabolism is believed to be affected by genetic factors [151]. Vitamin D has been found to be related to the epigenetic regulation of many genes. The presence of vitamin D receptor in beta cells conforms its relationship to the insulin secretion [152,153,154]. The knocking out of vitamin D receptor and hypovitaminosis D can impair insulin secretion, and treatment of vitamin D can induce insulin-dependent glucose uptake [145,146,155,156]. This shows that deficiency of vitamin D can cause insulin resistance.

### Strengths and Weaknesses

Systematic search strategy was used during this study which is the strength of this study. “Grading of Recommendations Assessment, Development and Evaluation (GRADE)” was used to determine of the quality of the studies. The range of 95% confidence interval was short showing the relevance of vitamin D in the correction of insulin resistance. The chance of residual confounding (due to a range of participants with different ages, skin type and exposure to sunlight) always exists in observational studies which is the weakness; however, the number of participants are always high in observational studies which is the strength of this study. The observational studies as compared to randomized controlled trials (RCTs), are not blinded and randomized which is the disadvantage in this case. Moreover, all studies did not give the exact information about vitamin D supplementation and the exposure time to sun which could be the source of confounding here. Taking in consideration all strengths and weaknesses the evidence is considered to be of moderate quality.

## 5. Conclusions

The meta-analysis shows that the status of vitamin D is inversely related to HOMA-IR. It was evident from the subgroup analysis that this correlation was intensely dependent on the BMI as it gets stronger with increasing BMI from lower BMI quartile to higher BMI quartiles. Therefore, we suggest that there is a part of vitamin D in the transcription of the insulin gene and secretion from beta cells which is highly dependent on the BMI. The female population found to be more dependent on the status of vitamin D for their insulin resistance level. A supplementation of vitamin D to the female population might have greater impact in lowering insulin resistance in female population compared to their male counterparts. The latitude and the methods used for the determination of vitamin D did not prove to have any effect on the association of vitamin D status and HOMA-IR as determined by meta-regression analysis. High-quality randomized controlled trials are needed to endorse the correlation between vitamin D status and HOMA-IR using different doses of vitamin D for a long time.

## References

[1] Bischoff-Ferrari H.A., Borchers M., Gudat F., Durmuller U., Stahelin H.B., Dick W. (2004). Vitamin D receptor expression in human muscle tissue decreases with age. J. Bone Miner. Res..

[2] Ku Y.C., Liu M.E., Ku C.S., Liu T.Y., Lin S.L. (2013). Relationship between vitamin D deficiency and cardiovascular disease. World J. Cardiol..

[3] Hahn S., Haselhorst U., Tan S., Quadbeck B., Schmidt M., Roesler S., Kimmig R., Mann K., Janssen O.E. (2006). Low serum 25-hydroxyvitamin D concentrations are associated with insulin resistance and obesity in women with polycystic ovary syndrome. Exp. Clin. Endocrinol. Diabetes.

[4] Yoon H., Kim G.S., Kim S.G., Moon A.E. (2015). The relationship between metabolic syndrome and increase of metabolic syndrome score and serum vitamin D levels in Korean adults: 2012 Korean National Health and Nutrition Examination Survey. J. Clin. Biochem. Nutr..

[5] Lionetti L., Mollica M.P., Lombardi A., Cavaliere G., Gifuni G., Barletta A. (2009). From chronic overnutrition to insulin resistance: The role of fat-storing capacity and inflammation. Nutr. Metab. Cardiovasc. Dis..

[6] Cruz K.J.C., Soares de Oliveira A.R., Pereira Pinto D., Silva Morais J.B., da Silva Lima F., Colli C., Torres-Leal F.L., do Nascimento Marreiro D. (2014). Influence of magnesium on insulin resistance in obese women. Biol. Trace Elem. Res..

[7] Greenfield J.R., Campbel L.V. (2004). Insulin resistance and obesity. Clin. Dermatol..

[8] Johnson A.R., Milner J.J., Makowski L. (2012). The inflammation highway: Metabolism accelerates inflammatory traffic in obesity. Immunol. Rev..

[9] Kahn S.E., Hull R.L., Utzschneider K.M. (2006). Mechanisms linking obesity to insulin resistance and type 2 diabetes. Nature.

[10] Qaid M.M., Abdelrahman M.M. (2016). Role of insulin and other related hormones in energy metabolism-A review. Cogent Food Agric..

[11] Ravier M.A., Rutter G.A. (2006). Glucose or insulin, but not zinc ions, inhibit glucagon secretion from mouse pancreatic alpha-cells. Diabetes.

[12] Williamson J.R., Browning E.T., Olson M. (1968). Interrelations between fatty acid oxidation and the control of gluconeogenesis in perfused rat liver. Adv. Enzym. Regul..

[13] Boucher B.J., John W.G., Noonan K. (2004). Hypovitaminosis D is associated with insulin resistance and β cell dysfunction. Am. J. Clin. Nutr..

[14] Saintonge H.B., Gerber L.M. (2009). Implications of a new definition of vitamin D deficiency in a multiracial US adolescent population: The National Health and Nutrition Examination Survey III. Pediatrics.

[15] Brancati F.L., Kao W.H.L., Folsom A.R., Watson R.L., Szklo M. (2000). Incident type 2 diabetes mellitus in African American and white adults: The atherosclerosis risk in communities study. J. Am. Med. Assoc..

[16] De Souza C.J., Meier A.H. (1987). Circadian and seasonal variations of plasma insulin and cortisol concentrations in the Syrian hamster, Mesocricetus auratus. Chronobiol. Int..

[17] Pittas A.G., Lau J., Hu F.B., Dawson-Hughes B. (2007). The role of vitamin D and calcium in type 2 diabetes. A systematic review and meta-analysis. J. Clin. Endocrinol. Metab..

[18] Marreiro D.N., Geloneze B., Tambascia M.A., Lerário A.C., Halpern A., Cozzolino S.M.F. (2004). Participação do Zinco na Resistência à Insulina. Arq. Bras. Endocrinol. Metabol..

[19] Salehpour A., Hosseinpanah F., Shidfar F., Vafa M., Razaghi M., Dehghani S., Gohari M. (2012). A 12-week double-blind randomized clinical trial of vitamin D3 supplementation on body fat mass in healthy overweight and obese women. Nutr. J..

[20] Rafiq S., Jeppesen P.B. (2018). Is hypovitaminosis D related to incidence of type 2 diabetes and high fasting glucose level in healthy subjects: A systematic review and meta-analysis of observational studies. Nutrients.

[21] Szlagatys-Sidorkiewicz A., Brzeziński M., Jankowska A., Metelska P., Słomińska-Frączek M., Socha P. (2017). Long-term effects of vitamin D supplementation in vitamin D deficient obese children participating in an integrated weight-loss programme (a double-blind placebo-controlled study)—Rationale for the study design. BMC Pediatr..

[22] Roth C.L., Elfers C., Kratz M., Hoofnagle A.N. (2011). Vitamin D deficiency in obese children and its relationship to insulin resistance and adipokines. J. Obes..

[23] Wamberg L., Pedersen S.B., Rejnmark L., Richelsen B. (2015). Causes of vitamin D deficiency and effect of vitamin D supplementation on metabolic complications in obesity: A review. Curr. Obes. Rep..

[24] Belenchia A.M., Tosh A.K., Hillman L.S., Peterson C.A. (2013). Correcting vitamin D insufficiency improves insulin sensitivity in obese adolescents: A randomized controlled trial. Am. J. Clin. Nutr..

[25] Nagpal J., Pande J.N., Bhartia A. (2009). A double-blind, randomized, placebocontrolled trial of the short-term effect of vitamin D3 supplementation on insulin sensitivity in apparently healthy, middle-aged, centrally obese men. Diabet. Med..

[26] Peterson C.A., Tosh A.K., Belenchia A.M. (2014). Vitamin D insufficiency and insulin resistance in obese adolescents. Ther. Adv. Endocrinol. Metab..

[27] Yousefi Rad E., Djalali M., Koohdani F., Saboor-Yaraghi A.A., Eshraghian M.R., Javanbakht M.H., Saboori S., Zarei M., Hosseinzadeh-Attar M.J. (2014). The effects of vitamin D supplementation on glucose control and insulin resistance in patients with diabetes type 2: A randomized clinical trial study. Iran. J. Public Health.

[28] Lazear J., Kapustin J. (2014). Vitamin D deficiency and type 2 diabetes: A retrospective review. J. Nurse Pract..

[29] Alvarez J.A., Bush N.C., Choquette S.S., Hunter G.R., Darnell B.E., Oster R.A., Gower B.A. (2010). Vitamin D intake is associated with insulin sensitivity in African American, but not European American, women. Nutr. Metab..

[30] Dutta D., Maisnam I., Shrivastava A., Sinha A., Ghosh S., Mukhopadhyay P., Mukhopadhyay S., Chowdhury S. (2013). Serum vitamin-D predicts insulin resistance in individuals with prediabetes. Indian J. Med. Res..

[31] Kabadi S., Lee B., Liu L. (2012). Joint effects of obesity and vitamin D insufficiency on insulin resistance and type 2 diabetes. Diabetes Care.

[32] Kim S., Lim J., Kye S., Joung H. (2012). Association between vitamin D status and metabolic syndrome risk among Korean population: Based on the Korean National Health and Nutrition Examination Survey IV-2, 2008. Diabetes Res. Clin. Pract..

[33] Kobzaa V.M., Feet J.C., Zhoua J., Conley T.B., Peacock M., Reger H.B.I., Palmad G.D., Campbell W.W. (2013). Vitamin D status and resistance exercise training independently affect glucose tolerance in older adults. Nutr. Res..

[34] Nguyen V.T., Li X., Elli E.F., Ayloo S.M., Castellanos K.J., Fantuzzi G., Freels S., Braunschweig C.L. (2015). Vitamin D, inflammation, and relations to insulin resistance in premenopausal women with morbid obesity. Obesity.

[35] Scragg R., Sowers M.R., Bell C. (2004). Serum 25-hydroxyvitamin D, diabetes, and ethnicity in the Third National Health and Nutrition Examination Survey. Diabetes Care.

[36] Weiler H.A., Lowea J., Krahn J., Leslie W.D. (2013). Osteocalcin and vitamin D status are inversely associated with homeostatic model assessment of insulin resistance in Canadian Aboriginal and white women: The First Nations Bone Health Study. J. Nutr. Biochem..

[37] Al-Sultan A.I., Amin T.T., Abou-Seif M.A., Al Naboli M.R. (2011). Vitamin D, parathyroid hormone levels and insulin sensitivity among obese young adult Saudis. Eur. Rev. Med. Pharmacol. Sci..

[38] Bilge U., Unalacak M., Unluoglu I., Ipek M., Celer O., Akalın A. (2015). Relationship between 1,25-dihydroxy Vitamin D levels and homeostatic model assessment insulin resistance values in obese subjects. Niger. J. Clin. Pract..

[39] Gedik O., Akalin S. (1986). Effect of vitamin D deficiency and repletion on insulin and glucagon secretion in man. Diabetologia.

[40] Justice J.N., Pierpoint L.A., Mani D., Schwartz R.S., Enoka R.M. (2014). Motor function is associated with 1,25(OH)2D and indices of insulin–glucose dynamics in non-diabetic older adults. Aging Clin. Exp. Res..

[41] Kabadi S.M., Liu L., Auchincloss A.H., Zakeri I.F. (2013). Multivariate path analysis of serum 25-hydroxyvitamin D concentration, inflammation, and risk of type 2 diabetes mellitus. Dis. Mark..

[42] Kayaniyil R.R., Harris S.B., Vieth R., Knight J.A., Gerstein H.C., Perkins B.A., Zinman B., Hanley A.J. (2011). Prospective associations of vitamin D with b-cell function and glycemia, the PROspective metabolism and ISlet cell evaluation (PROMISE) cohort study. Diabetes.

[43] Lee B.K., Park S., Kim Y. (2012). Age- and gender-specific associations between low serum 25-hydroxyvitamin D level and type 2 diabetes in the Korean general population: Analysis of 2008-2009 Korean National Health and Nutrition Examination. Survey data. Asia Pac. J. Clin. Nutr..

[44] Lu L., Wu Y., Qi Q., Liu C., Gan W., Zhu J., Li H., Lin X. (2012). Associations of type 2 diabetes with common variants in PPARD and the modifying effect of vitamin D among middle-aged and elderly Chinese. PLoS ONE.

[45] Marques-Vidal P., Vollenweider P., Guessous I., Henry H., Boulat O., Waeber G., Jornayvaz F.R. (2015). Serum vitamin D concentrations are not associated with insulin resistance in Swiss adults. J. Nutr..

[46] Renzaho A.M.N., Nowson C., Kaur A., Halliday J.A., Fong D., DeSilva J. (2011). Prevalence of vitamin D insufficiency and risk factors for type 2 diabetes and cardiovascular disease among African migrant and refugee adults in Melbourne. Asia Pac. J. Clin. Nutr..

[47] Scott D., Joham A., Teede H., Gibson-Helm M., Harrison C., Cassar S., Hutchison S., Ebeling P.R., Stepto N., de Courten B. (2016). Associations of vitamin D with inter- and intra-muscular adipose tissue and insulin resistance in women with and without polycystic ovary syndrome. Nutrients.

[48] Sorkin J.D., Vasaitis T.S., Streeten E., Ryan A.S., Goldberg A.P. (2014). Evidence for threshold effects of 25-hydroxyvitamin D on glucose tolerance and insulin resistance in black and white obese postmenopausal women. J. Nutr..

[49] Stadlmayr A., Aigner E., Huber-Schonauer U., Niederseer D., Zwerina J., Husar-Memmer E., Hohla F., Schett G., Patsch W., Datz C. (2015). Relations of vitamin D status, gender and type 2 diabetes in middle-aged Caucasians. Acta Diabetol..

[50] Tzotzas T., Papadopoulou F.G., Tziomalos K., Karras S., Gastaris K., Perros P., Krassas G.E. (2010). Rising serum 25-hydroxy-vitamin D levels after weight loss in obese women correlate with improvement in insulin resistance. J. Clin. Endocrinol. Metab..

[51] Vigna L., Cassinelli L., Tirelli A.S., Felicetta I., Napolitano F., Tomaino L., Mutti M., Barberi C.E., Riboldi L. (2017). 25(OH)D levels in relation to gender, overweight, insulin resistance, and inflammation in a cross- sectional cohort of northern Italian workers: Evidence in support of preventive health care programs. J. Am. Coll. Nutr..

[52] Vujosevic S., Borozan S., Radojevic N., Aligrudic S., Bozovic D. (2014). Relationship between 25-hydroxyvitamin D and newly diagnosed type 2 diabetes mellitus in postmenopausal women with osteoporosis. Med. Princ. Pract..

[53] Al-Shoumer K.A., Al-Asoosi A.A., Ali A.H., Nair V.S. (2013). Does Insulin Resistance in Type 2 Diabetes Alter Vitamin D Status?. Prim. Care Diabetes.

[54] Bellan M., Guzzaloni G., Rinaldi M., Merlotti E., Ferrari C., Tagliaferri A., Pirisi M., Aimaretti G., Scacchi M., Marzullo P. (2014). Altered glucose metabolism rather than naïve type 2 diabetes mellitus (T2DM) is related to vitamin D status in severe obesity. Cardiovasc. Diabetol..

[55] Dalgard C., Skaalum M., Weihe P.P., Grandjean P. (2011). DMSC vitamin D status in relation to glucose metabolism and type 2 diabetes in septuagenarians. Diabetes Care.

[56] Esteghamatia A., Aryanab B., Esteghamatia A., Nakhjavania M. (2014). Differences in vitamin D concentration between metabolically healthy andunhealthy obese adults: Associations with inflammatory and cardiometabolicmarkers in 4391 subjects. Diabetes Metab..

[57] Huang Y., Li X., Wang M., Ning H., Lima A., Li Y., Sun C. (2013). Lipoprotein lipase links vitamin D, insulin resistance, and type 2 diabetes: A cross-sectional epidemiological study. Cardiovasc. Diabetol..

[58] Jiang H., Peng S. (2014). The relationship between serum vitamin D and HOMA-IR in overweight elderly patients. Int. J. Cardiol..

[59] Park H.Y., Lim Y., Kim J.H., Bae S., Oh S., Hong Y. (2012). Association of serum 25-hydroxyvitamin D levels with markers for metabolic syndrome in the elderly: A repeated measure analysis. J. Korean Med. Sci..

[60] Kavadar G., Demircioglu D.T., Ozgonenel L., Emre T.Y. (2015). The relationship between vitamin D status, physical activity and insulin resistance in overweight and obese subjects. Bosn. J. Basic Med. Sci..

[61] Kim M.K., Kang M.I., Oh K.W., Kwon H.S., Lee J.H., Lee W.C., Yoon K., Ho Y. (2010). The association of serum vitamin D level with presence of metabolic syndrome and hypertension in middle-aged Korean subjects. Clin. Endocrinol..

[62] Nielsen N.O., Bjerregaard P., Rønn P.F., Friis H., Andersen S., Melbye M. (2016). Associations between Vitamin D status and type 2 diabetes measures among Inuit in Greenland may be affected by other factors. PLoS ONE.

[63] Pinelli N.R., Jaber L.A., Brown M.B., Herman W.H. (2010). 3serum 25-hydroxy vitamin D and insulin resistance, metabolic syndrome, and glucose intolerance among Arab Americans. Diabetes Care.

[64] Wright O.R.L., Hickman I.J., Petchey W.G., Sullivan C.M., Ong C., Rose F.J., Ng C., Prins J.B., Whitehead J.P., Moore-Sullivan T.M. (2013). The effect of 25-hydroxyvitamin D on insulin sensitivity in obesity: Is it mediated via adiponectin?. Can. J. Physiol. Pharmacol..

[65] Chonchol M., Cigolini M., Targher G. (2008). Association between 25-hydroxyvitamin D deficiency and cardiovascular disease in type 2 diabetic patients with mild kidney dysfunction. Nephrol. Dial. Transplant..

[66] Esteghamati A., Aryan Z., Esteghamati A.R., Nakhjavani M. (2015). Vitamin D deficiency is associated with insulin resistance in nondiabetics and reduced insulin production in type 2 diabetics. Horm. Metab. Res..

[67] Nama G.E., Kima D.H., Choa K.H., Parkb Y.G., Hanb K.D., Choi Y.S., Kim S.M., Ko B.J., Kim Y.H., Lee K.S. (2012). Estimate of a predictive cut-off value for serum 25-hydroxyvitamin D reflecting abdominal obesity in Korean adolescents. Nutr. Res..

[68] Al-Daghri N.M., Al-Attas O.S., Al-Okail M.S., Alkharfy K.M., Al-Yousef M.A., Nadhrah H.M., Sabico S.B., Chrousos G.P. (2010). Severe hypovitaminosis D is widespread and more common in non-diabetics than diabetics in Saudi adults. Saudi Med. J..

[69] Eraslan S., Kizilgul M., Uzunlulu M., Colak Y., Ozturk O., Tuncer I. (2013). Frequency of metabolic syndrome and 25-hydroxyvitamin D3 levels in patients with non-alcoholic fatty liver disease. Minerva Med..

[70] Hutchinson M.S., Figenschau Y., Almås B., Njølstad I., Jorde R. (2011). Serum 25-hydroxyvitamin D levels in subjects with reduced glucose tolerance and type 2 diabetes—The Tromsø OGTT-study. Int. J. Vitam. Nutr. Res..

[71] Imura H., Seino Y., Ishida H. (1985). Osteopenia and circulating levels of vitamin D metabolites in diabetes mellitus. J. Nutr. Sci. Vitaminol..

[72] Inomata S., Kadowaki S., Yamatani T., Fukase M., Fujita T. (1986). Effect of 1 alpha (OH)-vitamin D3 on insulin secretion in diabetes mellitus. Bone Miner..

[73] Mhatre M., Hall M. (2010). Student forum: Does calcium and vitamin D intake affect incidence of type 2 diabetes mellitus and insulin resistance syndrome?. Consult. Pharm..

[74] Mirzaei K., Hossein-Nezhad A., Keshavarz S.A., Eshaghi S.M., Koohdani F., Saboor-Yaraghi A.A., Hosseini S., Tootee A., Djalali M. (2014). Insulin resistance via modification of PGC1α function identifying a possible preventive role of vitamin D analogues in chronic inflammatory state of obesity. A double blind clinical trial study. Minerva Med..

[75] Abbasi F., Blasey C., Feldman D., Caulfield C.P., Hantash F.M., Reaven G.M. (2015). Low circulating 25-hydroxyvitamin D concentrations are associated with defects in insulin action and insulin secretion in persons with prediabetes. J. Nutr..

[76] Abdelkarem H.M., El-Sherif M.A., Gomaa S.B. (2016). Vitamin D status and insulin resistance among young obese Saudi females. Saudi Med. J..

[77] Al-Daghri N.M., Al-Attas O.S., Alokail M.S., Alkharfy K.M., Al-Othman A., Draz H.M., Yakout S.M., Al-Saleh Y., Al-Yousef M., Sabico S. (2013). Hypovitaminosis D associations with adverse metabolic parameters are accentuated in patients with Type 2 diabetes mellitus: A body mass index-independent role of adiponectin?. J. Endocrinol. Investig..

[78] Bhatt S.P., Misra A., Sharma M., Guleria R., Pandey R.M., Luthra K., Vikram N.K. (2014). Vitamin D insufficiency is associated with abdominal obesity in urban asian indians without diabetes in north india. Diabetes Technol. Ther..

[79] Bindal M.E., Taskapan H. (2011). Hypovitaminosis D and insulin resistance in peritoneal dialysis patients. Int. Urol. Nephrol..

[80] Boonchaya-anant P., Holick F.M., Apovian C.M. (2014). Serum 25-hydroxyvitamin D levels and metabolic health status in extremely obese individuals. Obesity.

[81] Botella-Carretero J.I., Alvarez-Blasco F., Villafruela J.J., Balsa J.A., Vazquez C., Escobar-Morreale H.F. (2007). Vitamin D deficiency is associated with the metabolic syndrome in morbid obesity. Clin. Nutr..

[82] Chacko S.A., Song Y., Manson J.E., Horn L.V., Eaton C., Martin L.W., McTiernan A., Curb J.D., Wylie-Rosett J., Phillips L.S. (2011). Serum 25-hydroxyvitamin D concentrations in relation to cardiometabolic risk factors and metabolic syndrome in postmenopausal women. Am. J. Clin. Nutr..

[83] Chung J., Hong S. (2013). Vitamin D status and its association with cardiometabolic risk factors in Korean adults based on a 2008-2010 Korean national health and nutrition examination survey nutrition research and practice. Nutr. Res. Pract..

[84] Coney P., Demers L.M., Dodson W.C., Kunselman A.R., Ladson G., Legro R.S. (2012). Determination of vitamin D in relation to body mass index and race in a defined population of black and white women. Int. J. Gynaecol. Obstet..

[85] Danziger J., Biggs M.L., Niemi M., Ix J.H., Kizer J.R., Djoussé L., de Boer I.H., Siscovick D.S., Kestenbaum B., Mukamal K.J. (2013). Circulating 25-hydroxyvitamin D is associated with insulin resistance cross-sectionally but not longitudinally in older adults: The Cardiovascular Health Study. Metab. Clin. Exp..

[86] De Pergola G., Nitti A., Bartolomeo N., Gesuita A., Giagulli V.A., Triggiani V., Guastamacchia E., Silvestris F. (2013). Possible role of hyperinsulinemia and insulin resistance in lower vitamin D levels in overweight and obese patients. Biomed. Res. Int..

[87] Del Gobbo L.C., Song Y., Dannenbaum D.A., Dewailly E., Egeland G.M. (2011). Serum 25-hydroxyvitamin D is not associated with insulin resistance or beta cell function in Canadian Cree. J. Nutr..

[88] Forouhi N.G., Luan J., Cooper A., Boucher B.J., Wareham N.J. (2008). Baseline serum 25-hydroxy vitamin D is predictive of future glycemic status and insulin resistance. Diabetes.

[89] Gannage-Yared M.L., Chedid R., Khalife S., Azzi E., Zoghbi F., Halaby G. (2009). Vitamin D in relation to metabolic risk factors, insulin sensitivity and adiponectin in a young Middle-Eastern population. Eur. J. Endocrinol..

[90] Grimnes G., Figenschau Y., Almas B., Jorde R. (2011). Vitamin D, insulin secretion, sensitivity, and lipids results from a case-control study and a randomized controlled trial using hyperglycemic clamp technique. Diabetes.

[91] Grineva E.N., Karonova T., Micheeva E., Belyaeva O., Nikitina I.L. (2013). Vitamin D deficiency is a risk factor for obesity and diabetes type 2 in women at late reproductive age. AGING.

[92] Ha C., Han T., Lee S., Cho J., Kang H. (2014). Association between serum vitamin D status and metabolic syndrome in Korean young men. Epidemiology.

[93] Hao Y., Ma X., Shen Y., Ni J., Luo Y., Xiao Y., Bao Y., Jia W. (2014). Associations of serum 25-hydroxyvitamin D3 levels with Visceral adipose tissue in Chinese men with normal glucose tolerance. PLoS ONE.

[94] Hjelmesæth J., Hofsø D., Aasheim E.T., Jenssen T., Moan J., Hager H., Røislien J., Bollerslev J. (2009). Parathyroid hormone, but not vitamin D, is associated with the metabolic syndrome in morbidly obese women and men: A cross-sectional study. Cardiovasc. Diabetol..

[95] Hong H.C., Lee J., Choi H.Y., Yang S.J., Yoo H.J., Seo J.A., Kim S.G., Kim N.H., Baik S.H., Choi D.S. (2013). Liver enzymes and vitamin D levels in metabolically healthy but obese individuals: Korean National Health and Nutrition Examination Survey. Metab. Clin. Exp..

[96] Huang C., Chang H., Lu C., Tseng F., Lee L., Huang K. (2015). Vitamin D status and risk of metabolic syndrome among non-diabetic young adults. Clin. Nutr..

[97] Kayaniyil S., Vieth R., Retnakaran R., Knight J., Qi Y., Gerstein H.C., Perkins B.A., Harris S.B., Zinman B., Hanley A.J. (2010). Association of vitamin D with insulin resistance and β-cell dysfunction in subjects at risk for type 2 diabetes. Diabetes Care.

[98] Lai S., Coppola B., Dimko M., Galani A., Innico G., Frassetti N., Mariotti A. (2014). Vitamin D deficiency, insulin resistance, and ventricular hypertrophy in the early stages of chronic kidney disease. Ren. Fail..

[99] Li H.W.R., Brereton R.E., Anderson R.A., Wallace A.M., Ho C.K.M. (2011). Vitamin D deficiency is common and associated with metabolic risk factors in patients with polycystic ovary syndrome. Metab. Clin. Exp..

[100] Liu E., Meigs J.B., Pittas A.G., McKeown N.M., Economos C.D., Booth S.L., Jacques P.F. (2009). Plasma 25-hydroxyvitamin D is associated with markers of the insulin resistant phenotype in nondiabetic adults. J. Nutr..

[101] Lu L., Yu Z., Pan A., Hu F.B., Franco O.H., Li H., Li X., Yang X., Chen Y., Lin X. (2009). Plasma 25-hydroxyvitamin D concentration and metabolic syndrome among middle-aged and elderly Chinese individuals. Diabetes Care.

[102] Moore A., Hochner H., Sitlani C.M., Williams M.A., Hoofnagle A.N., de Boer I.H., Kestenbaum B., Siscovick D.S., Friedlander Y., Enquobahrie D.A. (2015). Plasma vitamin D is associated with fasting insulin and homeostatic model assessment of insulin resistance in young adult males, but not females, of the Jerusalem Perinatal Study. Public Health Nutr..

[103] Muscogiuri G., Sorice G.P., Prioletta A., Policola C., Casa S.D., Pontecorvi A., Giaccari A. (2010). 25-hydroxyvitamin D concentration correlates with insulin-sensitivity and BMI in obesity. Obesity.

[104] O’Hartaigh B., Thomas G.N., Silbernagel G.N., Bosch J.A., Pilz S., Loerbroks A., Kleber M.E., Grammer T.B., Bohm B.O., Marz W. (2013). Association of 25-hydroxyvitamin D with type 2 diabetes among patients undergoing coronary angiography: Cross-sectional findings from the LUdwigshafen Risk and Cardiovascular Health (LURIC) Study. Clin. Endocrinol..

[105] Pham N.M., Akter S., Kurotani K., Nanri A., Sato M., Hayabuchi H., Yasuda K., Mizoue T. (2012). Serum 25-hydroxyvitamin D and markers of insulin resistance in a Japanese working population. Eur. J. Clin. Nutr..

[106] Sabio J.M., Vargas-Hitos J.A., Martinez-Bordonado J., Navarrete-Navarrete N., Chamorro-Diaz A.D., Olvera-Porcel C., Zamora M., Jimenez-Alonso J. (2015). Association between low 25-hydroxyvitamin D, insulin resistance and arterial stiffness in nondiabetic women with systemic lupus erythematosus. Lupus.

[107] Sheth J.J., Shah A., Sheth F.J., Trivedi S., Lele M., Shah N., Thakor P., Vaidya R. (2015). Does vitamin D play a significant role in type 2diabetes?. BMC Endocr. Disord..

[108] Song B.M., Rhee Y., Kim C.O., Youm Y., Kim K.M., Lee E.Y., Lee J.M., Yoon Y.M., Kim H.C. (2014). Urban-rural differences explain the association between serum 25-hydroxyvitamin D level and insulin resistance in Korea. Nutrients.

[109] Sun X., Cao Z., Tanisawa K., Ito T., Oshima S., Higuchi M. (2015). The relationship between serum 25-hydroxyvitamin D concentration, cardiorespiratory fitness, and insulin resistance in Japanese men. Nutrients.

[110] Tao M., Zhang Z., Yao-hua K.E., Jin-wei H.E., Wen-zhen F.U., Zhang C., Zhang Z. (2013). Association of serum 25-hydroxyvitamin D with insulin resistance and β-cell function in a healthy Chinese female population. Acta Pharmacol. Sin..

[111] Tosunbayraktar G., Bas M., Kut A., Buyukkaragoz A.H. (2015). Low serum 25(OH)D levels are assocıated to hıgher BMI and metabolic syndrome parameters in adults subjects in Turkey. Afr. Health Sci..

[112] Yin X., Sun Q., Zhang X., Lu Y., Sun C., Cui Y., Wang S. (2012). Serum 25(OH)D is inversely associated with metabolic syndrome risk profile among urban middle-aged Chinese population. Nutr. J..

[113] Holick M.F. (2005). Vitamin D: Important for prevention of osteoporosis, cardiovascular heart disease, type 1 diabetes, autoimmune diseases, and some cancers. South Med. J..

[114] dos Santos L.R., Lima A.G.A., Braz A.F., de Sousa Melo S.R., Morais J.B.S., Severo J.S., de Oliveira A.R.S., Cruz K.J.C., Marreiro D.d.N. (2017). Role of vitamin D in insulin resistance in obese individuals. Nutrire.

[115] Rafiq S., Jeppesen P.B. (2018). Body mass index, vitamin D, and type 2 diabetes: A systematic review and meta-analysis. Nutrients.

[116] Stein E.M., Strain G., Sinha N., Ortiz D., Pomp A., Dakin G., McMahon D.J., Bockman R., Silverberg S.J. (2009). Vitamin D insufficiency prior to bariatric surgery: Risk factors and a pilot treatment study. Clin. Endocrinol..

[117] Frayn K.N. (2001). Adipose tissue and the insulin resistance syndrome. Proc. Nutr. Soc..

[118] Mai S., Walker G., Vietti R., Cattaldo S., Mele C., Priano L., Marzullo P. (2017). Acute vitamin D3 supplementation in severe obesity: Evaluation of multimeric adiponectin. Nutrients.

[119] Entezari M., Khosravi Z., Kafeshani M., Tavasoli P., Zadeh A. (2018). Effect of Vitamin D supplementation on weight loss, glycemic indices, and lipid profile in obese and overweight women: A clinical trial study. Int. J. Prev. Med..

[120] Petersen K.F., Oral E.A., Dufour S., Befroy D., Ariyan C., Yu C., Cline G.W., DePaoli A.M., Taylor S.I., Gorden P. (2002). Leptin reverses insulin resistance and hepatic steatosis in patients with severe lipodystrophy. J. Clin. Investig..

[121] Marchesini G., Brizi M., Morselli-Labate A.M., Bianchi G., Bugianesi E., McCullough A.J., Forlani G., Melchionda N. (1999). Association of nonalcoholic fatty liver disease with insulin resistance. Am. J. Med..

[122] Dunaif A. (1997). Insulin resistance and the polycystic ovary syndrome: Mechanism and implications for pathogenesis. Endocr. Rev..

[123] Schwartz S.S., Epstein S., Corkey B.E., Grant S.F.A., Gavin I.J.R., Aguilar R.B., Herman M.E. (2017). A Unified pathophysiological construct of diabetes and its complications. Trends Endocrinol. Metab..

[124] Kasuga M. (2006). Insulin resistance and pancreatic beta cell failure. J. Clin. Investig..

[125] Kahn S.E. (2003). The relative contributions of insulin resistance and beta-cell dysfunction to the pathophysiology of type 2 diabetes. Diabetologia.

[126] Dattola A., Silvestri M., Bennardo L., Passante M., Scali E., Patruno C., Nistico S.P. (2020). Role of vitamins in skin health: A systematic review. Curr. Nutr. Rep..

[127] Seo J.A., Eun C.R., Cho H., Lee S.K., Yoo H.J., Kim S.G. (2013). Low vitamin D status is associated with non-alcoholic fatty liver disease independent of visceral obesity in Korean adults. PLoS ONE.

[128] Tomson J., Emberson J., Hill M., Gordon A., Armitage J., Shipley M. (2013). Vitamin D and risk of death from vascular and non-vascular causes in the whitehall study and meta-analyses of 12,000 Deaths. Eur. Heart J..

[129] Al Mheid I., Patel R.S., Tangpricha V., Quyyumi A.A. (2013). Vitamin D and cardiovascular disease: Is the evidence solid?. Eur. Heart J..

[130] Chiu K.C., Chu A., Go V.L.W., Saad M.F. (2004). Hypovitaminosis D is associated with insulin resistance and beta cell dysfunction. Am. J. Clin. Nutr..

[131] Oosterwerff M.M., Eekhoff E.M.W., Schoor N.M.V., Boeke A.J.P., Nanayakkara P., Meijnen R., Knol D.L., Kramer M.H.H., Lips P. (2014). Effect of moderate-dose vitamin D supplementation on insulin sensitivity in vitamin D–deficient non-Western immigrants in the Netherlands: A randomized placebo-controlled trial1–4. Am. J. Clin. Nutr..

[132] Kositsawat J., Freeman V., Gebber B., Geraci S. (2010). Association of A1c levels with vitamin D status in U.S. Adults. Diabetes Care.

[133] Hypponen E., Power C. (2006). Vitamin D status and glucose homeostasis in the 1958 British birth cohort: The role of obesity. Diabetes Care.

[134] Bril F., Maximos M., Portillo-Sanchez P., Biernacki D., Lomonaco R., Subbarayan S., Correa M., Lo M., Suman A., Cusi K. (2015). Relationship of vitamin D with insulin resistance and disease severity in non-alcoholic steatohepatitis. J. Hepatol..

[135] Chung S.J., Lee Y.A., Hong H., Kang M.J., Kwon H.J., Shin C.H., Yang S.W. (2014). Inverse relationship between vitamin D status and insulin resistance and the risk of impaired fasting glucose in Korean children and adolescents: The Korean National Health and Nutrition Examination Survey (KNHANES) 2009–2010. Public Health Nutr..

[136] Rafiq S., Jeppesen P.B. (2021). Insulin resistance is inversely associated with the status of vitamin D in both diabetic and non-diabetic populations. Nutrients.

[137] Barnard W.F., Saxena V.K., Wenny B.N., DeLuisi J.J. (2003). Daily surface UV exposure and its relationship to surface pollutant measurements. J. Air Waste Manag. Assoc..

[138] Elminir H.K. (2007). Sensitivity of ultraviolet solar radiation to anthropogenic air pollutants and weather conditions. Atmos. Res..

[139] Norman A.W., Frankel J.B., Heldt A.M., Grodsky G.M. (1980). Vitamin D deficiency inhibits pancreatic secretion of insulin. Science.

[140] Tanaka Y., Seino Y., Ishida M., Yamaoka K., Yabuuchi H., Ishida H., Seino S., Seino Y., Imura H. (1984). Effect of vitamin D3 on the pancreatic secretion of insulin and somatostatin. Acta Endocrinol..

[141] Kadowaki S., Norman A.W. (1984). Dietary vitamin D is essential for normal insulin secretion from the perfused rat pancreas. J. Clin. Investig..

[142] Clark S.A., Stumpf W.E., Sar M. (1981). Effect of 1,25 dihydroxyvitamin D3 on insulin secretion. Diabetes.

[143] Bourlon P.M., Faure-Dussert A., Billaudel B. (1999). The de novo synthesis of numerous proteins is decreased during vitamin D3 deficiency and is gradually restored by 1, 25-dihydroxyvitamin D3 repletion in the islets of langerhans of rats. J. Endocrinol..

[144] Maestro B., Davila N., Carranza M.C., Calle C. (2003). Identification of a Vitamin D response element in the human insulin receptor gene promoter. J. Steroid Biochem. Mol. Biol..

[145] Maestro B., Molero S., Bajo S., Davila N., Calle C. (2002). Transcriptional activation of the human insulin receptor gene by 1,25-dihydroxyvitamin D(3). Cell Biochem. Funct..

[146] Zeitz U., Weber K., Soegiarto D.W., Wolf E., Balling R., Erben R.G. (2003). Impaired insulin secretory capacity in mice lacking a functional vitamin D receptor. FASEB J.

[147] Mathieu C., Van Etten E., Gysemans C. (2001). In vitro and in vivo analysis of the immune system of vitamin D receptor knockout mice. J. Bone Miner. Res..

[148] Cade C., Norman A.W. (1987). Rapid Normalization/stimulation by 1,25-dihydroxyvitamin D3 of insulin secretion and glucose tolerance in the vitamin D-deficient rat. Endocrinology.

[149] Healy K.D., Frahm M.A., DeLuca H.F. (2005). 1,25-dihydroxyvitamin D3 up-regulates the renal vitamin D receptor through indirect gene activation and receptor stabilization. Arch. Biochem. Biophys..

[150] Alkharfy K.M., Al-Daghri N.M., Yakout S.M. (2012). Calcitriol Attenuates weight-related systemic inflammation and ultrastructural changes of the liver in a rodent model. Basic Clin. Pharmacol. Toxicol..

[151] Raffel L.J., Goodarzi M.O., Rotter J.I., Rimoin D.L., Connor J.M., Pyeritz R.E., Korf B. (1980). Diabetes mellitus. Principles and Practice of Medical Genetics.

[152] Wang Z.H., Shi X., Su H., Harshfield S., Gutin G.A., Snieder B., Dong H. (2013). A genome-wide methylation study of severe vitamin D deficiency in African American adolescents. J. Pediatr..

[153] Yu F., Cui L., Li X., Wang C., Ba Y., Wang L., Li J., Li C., Dai L., Li W. (2016). The genetic polymorphisms in vitamin D receptor and the risk of type 2 diabetes mellitus: An updated meta-analysis. Asia Pac. J. Clin. Nutr..

[154] Jeddi S., Syedmoradi L., Bagheripour F., Ghasemi A. (2015). The effects of vitamin D on insulin release from isolated islets of rats. Int. J. Endocrinol. Metab..

[155] Cade C., Norman A.W. (1986). Vitamin D3 improves impaired glucose tolerance and insulin secretion in the vitamin D-deficient rat in vivo. Endocrinology.

[156] Maestro B., Campion J., Davila N., Calle C. (2000). Stimulation by 1,25-dihydroxyvitamin D3 of insulin receptorexpression and insulin responsiveness for glucose transport in U-937 human promonocyticcells. Endocr. J..

